# Grandmaternal smoking during pregnancy is associated with differential DNA methylation in peripheral blood of their grandchildren

**DOI:** 10.1038/s41431-022-01081-2

**Published:** 2022-03-28

**Authors:** Sarah Holmes Watkins, Yasmin Iles-Caven, Marcus Pembrey, Jean Golding, Matthew Suderman

**Affiliations:** 1 University of Bristol, MRC Integrative Epidemiology Unit, Population Health Sciences, Bristol Medical School, Bristol, UK; 2 University of Bristol, Centre for Academic Child Health, Population Health Sciences, Bristol Medical School, Bristol, UK

**Keywords:** DNA methylation, Risk factors, Epidemiology

## Abstract

The idea that information can be transmitted to subsequent generation(s) by epigenetic means has been studied for decades but remains controversial in humans. Epidemiological studies have established that grandparental exposures are associated with health outcomes in their grandchildren, often with sex-specific effects; however, the mechanism of transmission is still unclear. We conducted Epigenome Wide Association Studies (EWAS) to test whether grandmaternal smoking during pregnancy is associated with altered DNA methylation (DNAm) in peripheral blood from their adolescent grandchildren. We used data from a birth cohort, with discovery and replication datasets of up to 1225 and 708 individuals (respectively, for the maternal line), aged 15–17 years, and tested replication in the same individuals at birth and 7 years. We show for the first time that DNAm at a small number of loci in cord blood is associated with grandmaternal smoking in humans. In adolescents we see suggestive associations in regions of the genome which we hypothesised a priori could be involved in transgenerational transmission - we observe sex-specific associations at two sites on the X chromosome and one in an imprinting control region. All are within transcription factor binding sites (TFBSs), and we observe enrichment for TFBS among the CpG sites with the strongest associations; however, there is limited evidence that the associations we see replicate between timepoints. The implication of this work is that effects of smoking during pregnancy may induce DNAm changes in later generations and that these changes are often sex-specific, in line with epidemiological associations.

## Introduction

The idea that information can be transmitted to subsequent generation(s) by epigenetic means remains controversial in humans [1]. The terminology used in the literature on this topic is not always consistent; here we use the term transgenerational to include all transmissions from one generation to subsequent generations. DNA methylation (DNAm) is a strong candidate for transgenerational epigenetic inheritance because it is heritable over cell division. A frequent argument against this is the two widespread phases of global de-methylation followed by re-methylation that all humans undergo in germ cells, and then immediately post-fertilization at the blastocyst stage (which allows cells to become pluripotent [2, 3]). However it has been shown that in human germ cells some genomic regions escape de-methylation [4], and imprinting control regions (ICRs) are not subject to de- or re-methylation in the early embryo [5]. Reports are also emerging of mechanisms that may preserve or restore DNAm at certain loci during the phase of germ cell de-methylation - such as transcription factors (TFs) [6, 7].

Most work on transgenerational responses in humans stems from epidemiological studies reporting associations between grandparental exposures or experiences and grandchild health outcomes. Effects are often sex-specific and unique to the maternal or paternal line [8, 9], which may suggest different mechanisms of transmission are responsible. Tobacco smoke exposure is frequently examined in transgenerational studies in humans because records of smoking behaviour are commonly available, and smoking behaviour is relatively easy to objectively and accurately recall and record, even by family members. Grandmaternal smoking during pregnancy has been associated with a number of health outcomes in their grandchildren: the paternal grandmother (PGM) smoking during pregnancy is associated with greater fat mass in their adult granddaughters, but not grandsons [10], and with reduced prevalence of myopia in their grandchildren up to 7 years of age (with associations between myopia before 7 years and DNAm at multiple loci) [11]. Maternal grandmother (MGM) smoking during pregnancy is associated with higher birth weight, and subsequent greater lean mass and higher cardiovascular fitness, in their grandsons [12, 13].

It has been well established that smoking is associated with differences in DNAm, both in the individual [14, 15] and in the offspring of mothers who smoke during pregnancy [16, 17]. One published study assessed the association of grandmaternal smoking with DNAm in their grandchildren, at 26 DNAm sites known to be associated with prenatal smoke exposure; none of these sites were found to be associated with grandmaternal smoking [18]. However, two studies testing associations genome-wide have found DNAm differences in the grandchildren of women exposed to lead [19] and violence [20] during pregnancy.

Here we test the hypothesis that grandmaternal smoking during pregnancy is associated with differences in DNAm in their grandchildren, at over 450,000 sites across the genome. We utilise the Avon Longitudinal Study of Parents and Children (ALSPAC) cohort [21], which has both DNAm data and detailed information about ancestral smoking. Almost 3000 methylomes have now been assayed for the index children at 15 years of age, making this the largest human cohort available to assess transgenerational epigenetic inheritance. We hypothesised a priori that effects were likely to be sex-specific in the grandchildren, and would be specific to maternal or paternal grandmothers, in line with previous transgenerational studies in humans [22]. We also hypothesised that differing mechanisms of transgenerational transmission would be evident for the maternal and paternal lines; if the MGM smoked during pregnancy, the ova that will form their grandchild would be directly exposed to effects of smoking, and so we would expect similar impact on DNAm as own smoking and maternal smoking during pregnancy. However, the PGM smoking would not directly expose the gamete that forms their grandchild, as her son will not produce sperm until puberty. Here any associations would have to be preserved in the father through to puberty, and as such we would expect them to differ to associations seen with direct exposure.

## Methods

### Cohort description

We used two DNAm datasets from the ALSPAC cohort; please see supplementary file for a detailed description of the ALSPAC cohort. Our discovery data were a newly generated dataset of 1869 individuals at 15–17 years of age, who had their methylomes assayed from peripheral blood samples on the Illumina EPIC Human Methylation microarray (EPIC array). Replication analyses utilised the original subsample of ALSPAC with DNAm data (known as the Accessible Resource for Integrated Epigenomic Studies, ARIES), assayed on the Illumina 450 K Human Methylation microarray (450k array) at birth (in cord blood), and at 7 and 15–17 years of age (in peripheral blood); 778 individuals in ARIES have measurements at all three timepoints [23]. No participants overlap between the discovery dataset and any of the replication datasets. Although replication is ideally conducted in a separate cohort, we could identify no other available DNAm datasets of a similar age with ancestral smoking data.

### Ancestral smoking data

We determined whether the maternal and paternal grandmothers of the ALSPAC study children smoked during pregnancy using questionnaires completed by the study mother and father. Maternal and paternal lines were tested separately. The smoking variable we used was a categorical ‘Yes’ or ‘No’; see supplementary file for details on how this was created.

### Study exclusions

To attempt to detect only grandmaternal effects, we excluded all individuals whose mother reported smoking whilst pregnant, and all adolescents who reported smoking themselves. We also excluded a small number of individuals from the EPIC dataset who were of non-white ethnicity, as reduced rates of both MGM and PGM smoking were associated with non-white ethnicity; please see supplementary file for details.

#### New ALSPAC methylomes assayed on EPIC array

DNA methylation profiles were generated for 1885 participants as previously described but using the Illumina Infinium MethylationEPIC Beadchip (EPIC array) rather than the Illumina Infinium HumanMethylation450k Beadchip (450k array) [24]. Genomic coordinates for probe targets on both the 450k and EPIC arrays refer to the GRCh37/hg19 genome build. Briefly, following DNA extraction, DNA was bisulfite converted using the Zymo EZ DNA Methylation^TM^ kit (Zymo, Irvine, CA), and DNA methylation was measured using EPIC arrays. Arrays were scanned using Illumina iScan, and the initial quality review was carried out using GenomeStudio. A wide range of batch variables was recorded in a purpose-built laboratory information management system (LIMS). Additional quality control and normalization were carried out using the *meffil* R package version 1.1.0 [24]. Of 1885 initial samples, 16 were found to have an unacceptably high proportion of undetected probes (proportion > 10% with detection *p*-value > 0.01). The remaining 1869 were normalized using functional normalization [25] as implemented in *meffil* with quantiles adjusted using 20 control probe principal components and slide as a random effect.

#### Original ARIES DNAm data assayed on 450k array

921 samples were available for the 15–17-year-olds from the original ARIES DNAm dataset, along with 849 individuals at birth (with samples from cord blood), and 910 individuals at 7 years (samples from peripheral blood). Consent for biological samples was collected in accordance with the Human Tissue Act (2004). Processing, extraction, and quality control of DNAm data has been described in detail for these samples [23], as have normalisation and outlying sample removal procedures [24]. Included in our numbers is the removal of 21 additional individuals as they were the only sample on a slide, preventing their adjustment for slide effects.

#### Filtering DNAm sites

All sites on the X and Y chromosomes were removed from the analyses using all individuals; the Y chromosome was removed from the single sex analyses. No other sites were removed, but results were checked against probes flagged as being cross-reactive or having a SNP at the CpG site, in the single base extension, or in the probe body [26].

### EWAS

Six EWAS were performed in both the discovery (EPIC) and replication (450k) datasets, testing the association of DNAm with MGM smoking in all individuals, in females, and in males, and with PGM smoking, in all individuals, in females, and in males. EWAS were conducted using *meffil*, which uses linear regression to assess CpG-trait associations [24]. For all EWAS with both sexes we included only the autosomes; for all single-sex EWAS we included sex chromosomes. A genome-wide significance threshold of *p* < 9e−08 was used for the EPIC analyses [27], and *p* < 2.4e−07 for the 450k [28]. Covariates for all EWAS were: age at DNAm sample; sex (for the analyses with both sexes); batch effects (plate for the EPIC samples, slide for the 450k samples); and cell count proportions estimated using a deconvolution algorithm [29] implemented in meffil, based on the ‘blood gse35069 complete’ cell type reference. Because known covariates can be imperfect and miss sources of unwanted variation, we conducted a sensitivity analysis adjusting for surrogate variables (using surrogate variable analysis (SVA) [30] as implemented in meffil) where we assessed correlation between effect sizes of the SVA and known covariates models. As there was high correlation between effect sizes (>0.97) the known covariates model was used for all analyses – the high correlation suggests that the main model accounted for all substantial sources of DNAm variation, and SVA risks removing biologically interesting sources of variation in the data.

### Testing for replication

We used three complementary approaches to test for replication of the sites most strongly associated with the exposure in the discovery dataset, as no single measure can capture this. Firstly, we took the 25 top associated sites from the EPIC analyses that were also present on the 450k array and assessed them for association in the 450k analyses at the equivalent of *p* < 0.05/25. Secondly, we correlated effect sizes (using Pearson correlation) between the discovery and replication datasets, for the top 10, 25, 50, 100, and 200 sites identified in each discovery EWAS. Finally, we conducted a binomial test for each discovery EWAS to ascertain whether the top 10, 25, 50, 100, and 200 sites replicated at *p* < 0.05 with the same direction of effect.

### Meta-analysis

For each of the six EWAS (MGM smoking: all individuals, males, and females; and PGM smoking: all individuals, males, and females), we meta-analysed results from the EPIC and 450k analyses at 15–17 years, using all sites common to both arrays. We performed meta-analysis of the effect sizes and standard errors using METAL [31].

### Lookup of associations in a priori specified genomic locations

We hypothesised that DNAm was most likely to associate with grandmaternal smoking during pregnancy in genomic regions specified by previous transgenerational work. We hypothesised associations on the X chromosome as X-inactivation is sex-specific and previous work has reported that associations between ancestral exposures and health outcomes in later generations are often sex-specific. We, therefore, tested this hypothesis by testing associations at all sites on the X chromosome separately in males and females using the X-specific Bonferroni threshold p < 2.7e–06. As some DNAm sites have been shown to escape the wave of de-methylation in germ cells [4], we tested associations at all CpG sites on the EPIC array (*n* = 36,051) belonging to the 116,618 regions of the genome that have been identified as escaping de-methylation [4] (which have recently been made available [32]) at the corresponding Bonferroni threshold *p* < 1.4e–6. Finally, as ICRs are not subject to the phases of de-methylation and re-methylation in the early embryo, we tested associations at the 984 CpG sites present on the EPIC array that belong to ICRs [33] (29 of which overlapped with the escapees) at the corresponding Bonferroni threshold *p* < 5.1e–5.

### Testing replication earlier in life

To ascertain whether any sites associated with grandmaternal smoking at 15–17 years are differentially methylated from birth, we repeated each EWAS using DNAm profiles for ALSPAC participants from blood samples collected at birth and 7 years (see supplementary table 1 for participant numbers). We included the same covariates as for the adolescents, aside from at birth where gestational age was substituted for age. As the birth and 7 years DNAm profiles were assayed from different sample types (blood spots and white cells at birth; white cells and whole blood at 7 years), sample type was also included as a covariate. In addition to using this analysis to assess replication of associations in the 15–17-year-olds, we assessed the opposite, replication of associations at the birth and age 7 in the 15–17-year-old discovery dataset.

### Transcription factor binding site (TFBS) enrichment analysis

To test the hypothesis that differential DNAm associated with grandmaternal smoking might be mediated by TFs preserving or maintaining methylation status, we tested whether DNAm sites were located near TFBS more than expected by chance. To do this we took the top 25 sites from each discovery EWAS and tested them for TFBS enrichment against all sites on the EPIC array used in our EWAS (n sites = 838,019) using LOLA locus overlap [34]. We used the Encode TFBS [35, 36] region set created by the LOLA team, comprising ChIP-seq data on 161 TFs (available through http://lolaweb.databio.org). We tested 100 bp on either side of the DNAm site, removing overlapping sites to prevent inflation of results. Results were reduced to TFBS measured in blood which were associated in at least one EWAS at *p* < 0.05. To assess whether individual sites identified in the main analysis were associated with a TFBS, we used the hg19 version of the UCSC genome browser [37]; https://genome-euro.ucsc.edu/.

### Enrichment of prenatal- and own smoking-associated sites

We tested the hypothesis that DNAm sites that are established as being associated with prenatal smoking and own smoking would be enriched in our EWAS associations, to ascertain whether transgenerational transmission might be related to these sites. To do this we evaluated statistical inflation of EWAS associations among the 568 DNAm sites (of which 540 were available on the EPIC array) previously reported to be associated with maternal prenatal smoking in cord blood [17], and the 2623 sites (2445 available on the EPIC array) reported to be associated with own smoking [38]. For each, inflation beyond expected levels was evaluated by generating QQ plots and lambda values. We then used a one-sided Wilcoxon rank-sum test to ask if DNAm sites associated with prenatal- and own-smoking had lower *p*-values in our EWAS than expected from a random selection.

### Enrichment of lean mass-associated sites

We finally sought to identify whether DNAm sites associated with grandmaternal smoking might be related to lean mass (a previously reported epidemiological association [13]). Although no published EWAS of lean mass is available, 47 sites associated with lean mass in the mothers in ALSPAC at *p* < 1e–04 are available in the EWAS Catalog [39]; http://www.ewascatalog.org/. We checked for inflation of these sites in our data using QQ plots and lambda values, and tested enrichment for these sites using a Wilcoxon rank sum test.

## Results

### Study characteristics

Of the 1869 individuals with EPIC array DNAm profiles passing QC, we removed 267 because they were either of non-white ethnicity or had missing ethnicity data, because non-white ethnicity was associated with lower rates of smoking for both MGM and PGM (*p* = 0.03 and 0.007, respectively). Of the remaining 1602 participants, 285 were removed because their mother reported smoking during her pregnancy, and 73 because they reported smoking themselves. Of the 910 individuals with 450k DNAm data passing QC and filtering, we removed 125 individuals because their mother reported smoking during pregnancy, and 59 as they reported smoking themselves. All individuals in the 450k dataset were of white ethnicity. Table 1 summarises the characteristics of the adolescent datasets; supplementary table 1 details the numbers of participants with complete data in each EWAS.

**Table 1:** Summary of variables used in the analysis for participants with DNAm data assayed on the EPIC and 450k arrays. MGM = maternal grandmother, PGM = paternal grandmother.

Dataset	Measure	MGM analysis	PGM analysis	Full cohort
EPIC (discovery)	N participants	1225	1021	1869
	Grandmaternal smoking (% yes)	30.90%	38.40%	34% MGM; 39% PGM^a^
	Sex (% Female)	50.70%	51.60%	52.40%
	Age (mean(SD))	17.8 (0.4)	17.8 (0.4)	17.8 (0.5)
450k (replication)	N participants	708	601	910
	Grandmaternal smoking (% yes)	29.90%	36.60%	31.7% MGM; 39.4% PGM^a^
	Sex (% Female)	51.10%	51.60%	51.60%
	Age (mean(SD))	17.1 (1)	17.1 (1)	17.1 (1)

^a^percentages in this cell are calculated after omitting participants with missing ancestral smoking data.

### Discovery EWAS results

No associations survived the Bonferroni-adjusted p-value threshold (*p* < 9e–8) in the EPIC array dataset. All associations *p* < 1e–04 using the main model are in supplementary table 2.

### Replication

All associations in the replication dataset (the samples assayed on the 450k array) at *p* < 1e–04 using the main model are reported in supplementary table 2. Firstly we tested replication at *p* < 0.05/25 for each of the six EWAS. For MGM smoking, the association at a single site on the X chromosome replicates in the females only analysis (cg19782749, *p* = 0.001; Table 2), although this does not survive adjustment for testing replication over the six EWAS we conducted (a threshold of p < 0.05/(6x25) = *p* < 0.0003). For PGM smoking, none of the associations at the top 25 sites replicate. Secondly, we evaluated correlation of effect sizes for associations at the top sites in each EWAS between the discovery and replication datasets, and here we do not find consistent evidence of replication in any of the six analyses. For MGM smoking in the all-individuals analysis, there were moderate negative correlations for the top 25 to 200 sites (*R* = –0.18 to –0.45, *p* < 0.03). Within each of the other five EWAS analyses, moderate correlations (*R* = 0.28 to 0.45, *p* < 0.05) were found for at most two thresholds- correlations were otherwise small (*R* < 0.2), and four analyses featured both negative and positive correlations. Thirdly, we asked if direction of effect was preserved in replication data for the top associated sites. There was again evidence supporting replication only for MGM smoking in female grandchildren (in four of five tests *p* < 0.009, binomial test), but none of the other EWAS. Details are in supplementary table 3.

**Table 2:** DNAm sites which were found to associate with grandmaternal smoking in all analyses. Models were adjusted for all covariates (age/gestational age, predicted cell counts, and batch effects).

Age (subset)	Array	Analysis	N	CpG	Chr	position	*p* value	Effect size
15–17 (females)	EPIC	MGM (replication)	621	cg19782749	X	132091720	5.20E−05	0.002
15–17 (females)	450k	MGM (replication)	362	cg19782749	X	132091720	0.001	0.001
15–17 (males)	EPIC	PGM (X chr)	494	cg27456137	X	129403024	1.90E−06	−0.01
15–17 (all individuals)	EPIC	PGM (ICR)	1021	cg15068552	7	130130203	2.20E−05	−0.02
Birth (all individuals)	450k	MGM EWAS	709	cg19426678	12	117537404	2.10E−07	−0.002
Birth (females)	450k	PGM EWAS	290	cg22682200	10	120001266	2.30E−09	−0.05
Birth (females)	450k	PGM EWAS	290	cg26827966	5	172189374	5.90E−08	−0.03

Chromosomal positions refer to the GRCh37/hg19 genome build.

Using the same replication methods, we evaluated agreement between associations observed in male and female stratified analyses in the discovery dataset. For MGM smoking, effects of associations at top female sites appear to be negatively correlated with effects in males (*R* = −0.21 to −0.43, *p* < 0.05). None of the other replication analyses yielded evidence for agreement or disagreement between top male and female associations. For PGM smoking, effect sizes of top female sites were positively correlated with effects in males (*R* = 0.34 to 0.66, *p* < 0.04); we also observe agreement in direction of effect for associations at the top 200 female sites. Details are in supplementary table 4.

### Meta-analysis

When meta-analysing the discovery (EPIC) and replication (450k) datasets, no associations across the 438,459 sites survived Bonferroni-adjustment for multiple tests (*p* < 2.4e−07).

### Lookup of associations in a priori specified genomic locations

Only one association on the X chromosome survived Bonferroni adjustment for the number of X chromosome sites (*p* < 2.7e−6). The association was with PGM smoking in males (cg27456137; *p* = 1.9e−06); Table 2. The probe for this site has been flagged [26] as cross-hybridising to a 49 bp sequence 500 bp from cg27456137, in which three probes on the EPIC array reside; though none were associated with either grandmother smoking near genome-wide significance (all *p* > 0.03). No sites within escapee regions were associated with grandmaternal smoking at the Bonferroni corrected *p*-value *p* < 1.5e−06 in the discovery dataset. We observe one association in an ICR that survives correction for multiple tests (*p* < 5.1e−05); the association is with PGM smoking (cg15068552, *p* = 2.2e−05); Table 2.

### Testing associations and replication earlier in life

In cord blood, we find one site associated with MGM smoking in all individuals, and two sites associated with PGM smoking in females (see Table 2 for a summary). In the 7-year-olds, no sites were associated with either grandmother smoking in any of the six analyses. All associations *p* < 1e−04 using the main model are reported in supplementary table 2. None of these associations were observed at adolescence (i.e., in the main discovery dataset) below the *p* < 0.05/3 threshold (all *p* > 0.07). We then tested whether two of the three associations observed at adolescence (i.e., in the discovery dataset) replicated at birth and at 7 years (cg15068552 in all individuals when the PGM smoked and cg19782749 in females when the MGM smoked; cg27456137 is not measured by the 450k array). We see a suggestion of replication at cg15068552 at birth in all individuals when the PGM smoked (*p* = 0.02), and at cg19782749 at 7 years in females when the MGM smoked (*p* = 0.04).

### Transcription factor binding site analysis

**Fig. 1:**
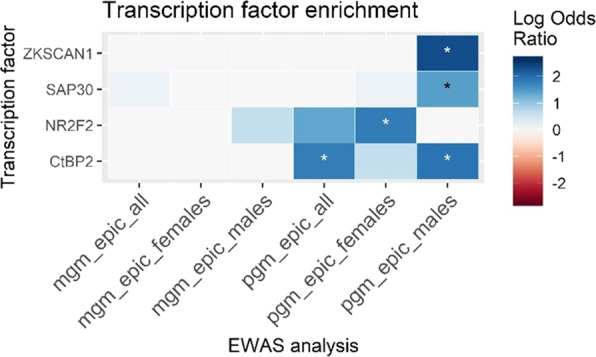
TFBS enrichments for TFBS that reached p < 0.05 for at least one of the six main discovery EWAS. Heatmap is coloured by the log odds ratio, * = *p* < 0.05. MGM = maternal grandmother, PGM = paternal grandmother.

Using locus overlap enrichment analysis (LOLA), we find enrichment of the top 25 EWAS associations at the TFBS for four TFs (nominal *p* < 0.05) in EWAS of PGM smoking. CtBP2 is enriched for the EWAS of males and females (log OR = 1.8, *p* = 0.02), although this may be driven by enrichment in the males; NR2F2 is enriched in the females-only EWAS (log OR = 1.8, p = 0.02); and CtBP2 (log OR = 1.9, p = 0.01), SAP30 (log OR = 1.4, *p* = 0.04), and ZKSCAN1 (log OR = 2.3, *p* = 0.02) are enriched in the males-only EWAS. There are no enrichments in the MGM smoking analyses. These enrichment results are illustrated in Fig. 1. We then used the UCSC genome browser to assess whether the six individual sites identified were located within TFBS. We find all six are located in sites that bind at least one TF; these are detailed in supplementary table 5.

### Enrichment of prenatal- and own smoking- associated sites

Among sites associated with prenatal smoking, we observe some inflation for associations with PGM smoking in males (lambda = 1.27 ± 0.13) and females (lambda = 1.12 ± 0.11). This inflation is replicated only for males in the 450k dataset (lambda = 1.46 ± 0.12). Among sites associated with own smoking, there is weak inflation for associations with PGM smoking in females (lambda = 1.16 ± 0.05), but this association is not replicated. Inflation results are summarised in Table 3.

**Table 3:** Summary of lambda values to illustrate inflation, and Wilcoxon rank-sum p-values to illustrate enrichment, of DNAm sites previously identified to be associated with prenatal smoke exposure and own smoking.

EWAS	Prenatal smoking sites				Own smoking sites			
	EPIC		450k		EPIC		450k	
	Lambda ± SE	Wilcoxon p-value	Lambda ± SE	Wilcoxon *p*-value	Lambda ± SE	Wilcoxon *p*-value	Lambda ± SE	Wilcoxon *p*-value
**MGM all**	0.93 ± 0.09	0.67	0.89 ± 0.09	0.88	0.96 ± 0.04	0.85	1.03 ± 0.04	0.2
**MGM females**	0.79 ± 0.07	0.91	1.08 ± 0.08	0.86	0.9 ± 0.04	0.53	1.01 ± 0.04	1
**MGM males**	0.93 ± 0.08	0.88	0.88 ± 0.09	**0.02**	0.89 ± 0.04	1	0.96 ± 0.05	**4.10E**−**05**
**PGM all**	**1.27** ± **0.11**	0.62	1.09 ± 0.11	**2.20E**−**05**	**1.21** ± **0.05**	0.86	0.97 ± 0.03	**1.10E**−**07**
**PGM females**	**1.12** ± **0.11**	0.58	0.85 ± 0.06	0.11	**1.16** ± **0.05**	0.09	0.81 ± 0.04	**0.01**
**PGM males**	**1.27** ± **0.13**	**0.02**	**1.46** ± **0.12**	**8.30E**−**08**	0.93 ± 0.05	0.99	**1.1** ± **0.05**	**1.42E**−**06**

Bold values indicate where inflation/enrichment is observed.

### Inflation test for lean mass-associated DNAm sites

We observe no evidence for inflation among CpG sites associated with lean mass. These inflation results are summarised in supplementary table 6.

## Discussion

In summary, we find suggestive evidence for effects of grandmaternal smoking during pregnancy on DNA methylation in her adolescent grandchildren in regions we hypothesised a priori; on the X chromosome, in an ICR, in TFBS, and among prenatal smoking-associated DNAm sites. However there is limited evidence of replication of associations, and the associations we do see would not survive correction for multiple tests. We find three associations in cord blood, but see limited evidence of associations persisting over time. In most cases, associations appear to be sex-specific in line with previous research [8–10].

The suggestive associations we see in adolescence indicate that some of the genomic regions we hypothesised a priori may be involved in the transmission of transgenerational responses. Two of the six sites we identify are on the X chromosome, giving a possible route by which sex-specific differences in transmission of responses across generations might occur. We find evidence for a single site residing within an ICR, but find no evidence for sites in regions known to escape de-methylation in germ cells. We find evidence suggesting TFs could have a role in the transmission of epigenetic responses to smoking across generations—both from the enrichment analysis, and the location of all six individual sites within TFBS. This is the most consistent line of evidence in our study and we suggest TFs may present the most promising line for future research. TF binding events could either shield DNAm from being modified in early development or induce DNAm changes consistent with ancestral smoking, as DNAm status can be restored by TFs during germline and embryonic development following erasure [6, 7]. Finally, we find suggestive evidence of replication of two sites identified in adolescents in earlier DNAm samples (one at birth and one at 7 years), although no site replicates across all three timepoints.

The limited evidence of persistence of association may suggest that DNAm is not the primary mechanism of transmission across generations. The associations that we see change over time, which is consistent with epidemiological observations that associations between grandmaternal smoking during pregnancy and grandchild outcomes are rarely observed at birth, and become stronger as the grandchild ages (particularly in late adolescence and early adulthood) [22]. As such it may be that we find differences at these DNAm sites due to another factor that is influenced by or associated with grandmaternal smoking, such as parental behaviour, or parental development being altered by the direct effect of prenatal smoking.

We find evidence of inflation and enrichment of sites associated with prenatal smoking only in males when their PGM smoked, and do not find consistent inflation of sites associated with own smoking; suggesting grandmaternal smoking may affect DNAm via different mechanisms than maternal smoking. The enrichment in the paternal rather than maternal line is in contrast to our hypothesis; it is the paternal line demonstrating effects similar to direct exposure, which again may suggest mechanisms other than direct epigenetic inheritance. The inflation we see in males is contrary to previous null prenatal findings [18]; this may be because we test a larger number of sites. We do not see any inflation or enrichment of lean mass-associated DNAm sites in our analyses, suggesting that differences in lean mass observed previously [13] may not be related to DNAm.

Strengths of our study are that we assessed grandmaternal smoking effects in a large cohort of humans with ancestral smoking data, alongside rich phenotypic data. We have DNAm data from birth so were able to assess whether DNAm differences at these sites are present between birth and adolescence. Limitations include that the 450k and EPIC array platforms only cover around 2% and 4% of the genome, respectively, and that our replication dataset came from the same birth cohort as the discovery data. Additionally, we note that the availability of grandchild DNAm data only for live offspring births in ALSPAC could induce bias in our findings since smoking decreases fertility and increases risk of miscarriage.

## Supplementary information

Supplementary file

## Data Availability

The data that supports the findings of this study are available from ALSPAC but restrictions apply to the availability of these data, which were used under license for the current study, and so are not publicly available. ALSPAC fully supports Wellcome and the RCUK policies on open access. The process for obtaining access to data is described on the study website: http://www.bristol.ac.uk/alspac/researchers/data-access/. The ALSPAC study website contains details of all the data that are available through a fully searchable data dictionary: http://www.bristol.ac.uk/alspac/researchers/our-data/.
